# 3-Hydroxystearic acid promotes cholesterol efflux and attenuates atherosclerosis via the ALKBH5/PAX-8/ABCA1 pathway

**DOI:** 10.3389/fimmu.2026.1750021

**Published:** 2026-01-30

**Authors:** Qin-Yi Zhou, Wang Liu, Zhen-Wang Zhao, Duo Gong, Xiao-Feng Ma, Chao-Ke Tang

**Affiliations:** 1 Key Laboratory for Arteriosclerology of Hunan Province, Institute of Cardiovascular Disease, Department of Cardiology, The Affiliated Nanhua Hospital, Hengyang Medical School, University of South China, Hengyang, Hunan, China; 2 Department of Gastrointestinal Surgery, The Affiliated Nanhua Hospital, Hengyang Medical School, University of South China, Hengyang, Hunan, China; 3 School of Basic Medicine, Health Science Center, Hubei University of Arts and Science, Xiangyang, Hubei, China

**Keywords:** 3-Hydroxystearic acid, atherosclerosis, ATP binding cassette transporter A1, cholesterol efflux, paired box protein 8, α-Ketoglutarate dependent dioxygenase alk B homolog 5

## Abstract

**Introduction:**

Atherosclerosis can trigger various cardiovascular and cerebrovascular diseases with complex pathogenesis. Macrophage proliferation, inflammatory responses, and lipid phagocytosis, which induce foam cell formation and accumulation, are critical in the development of early atherosclerotic lesions. The role of 3-Hydroxystearic acid (C18-3OH), a recently identified gut microbiota-derived metabolite, in atherosclerosis has not yet been clarified. This study aimed to investigate the role of the ALKBH5/PAX-8/ABCA1 pathway in C18-3OH-mediated regulation of macrophage cholesterol efflux and atherosclerosis and explore novel mechanisms of ABCA1 regulation from the perspective of m6A modification.

**Methods:**

RT-qPCR and Western blotting were used to detect gene and protein expression, respectively. ChIP-Seq was used to screen PAX-8 target genes, and ChIP-qPCR was used to validate PAX-8 binding to ABCA1. The SRAMP platform was used to predict m6A modification sites in PAX-8 mRNA sequences. Methylated RNA immunoprecipitation-qPCR (MeRIP-qPCR) was used to measure m6A modification levels of PAX-8 mRNA in foam cells. UHPLC-OEMS untargeted metabolomics were used to analyze differential fatty acid metabolites in an atherosclerotic mouse model. Specific kits were used to detect serum liver function markers (aspartate transaminase, AST; alanine aminotransferase, ALT), renal function markers (serum creatinine, Scr; blood urea nitrogen, BUN), and lipid profiles (HDL-C, TG, LDL-C, TC). Aortic sinus sections were prepared, and H&E, Oil Red O, and Masson staining were used to evaluate atherosclerotic plaques.

**Results:**

The results demonstrated that C18-3OH promoted cholesterol efflux in foam cells and alleviated lipid accumulation by upregulating ABCA1 expression. C18-3OH inhibited ALKBH5, increased PAX-8 mRNA m6A modification and PAX-8 expression, and upregulated ABCA1 to enhance cholesterol efflux. Serum metabolomics revealed reduced C18-3OH levels in high-fat diet-fed apoE-/- atherosclerotic mice. C18-3OH suppressed aortic ALKBH5 expression, elevated m6A modification of PAX-8 mRNA, and increased PAX-8 and ABCA1 expression. Furthermore, C18-3OH improved lipid metabolism and reduced the atherosclerotic plaque area in apoE-/- mice.

**Discussion:**

This study clarifies the impact and mechanisms of gut microbiota-derived C18-3OH on atherosclerosis progression, providing novel strategies for the precise prevention and treatment of atherosclerosis.

## 1 Introduction

Atherosclerosis constitutes the underlying pathological basis of cardiovascular diseases. Foam cells, characterized by cholesterol ester-enriched lipid droplets, represent a hallmark structure of atherosclerotic plaques (1). Macrophages take up modified lipoproteins, leading to intracellular lipid droplet accumulation and transformation into cholesterol-laden foam cells (2). The aggregation of foam cells within the vascular wall forms necrotic cores, which secrete many cytokines, exacerbate vascular inflammation and lipid deposition, and ultimately propagate atherosclerotic progression (3). Therefore, maintaining lipid metabolic homeostasis and reducing foam cell formation are critical therapeutic strategies for atherosclerosis.

Reverse cholesterol transport (RCT) refers to the process by which cholesterol is effluxed from cells in peripheral tissues, transported to the liver via high-density lipoprotein (HDL), and ultimately excreted through bile acid metabolism into feces (4). Macrophage-mediated RCT represents the first and most critical step in this pathway, serving as a key mechanism for reducing macrophage lipid accumulation and attenuating atherosclerotic progression (5). Enhancing macrophage cholesterol efflux and RCT efficiency can attenuate atherosclerotic progression and lower cardiovascular risks (6). ATP-binding cassette transporter A1 (ABCA1), a pivotal cellular cholesterol transporter expressed in macrophages, plays an essential role in initiating RCT (7, 8). ABCA1 facilitates the transport of intracellular free cholesterol (FC) to apolipoprotein A-I (apoA-I), forming nascent HDL particles (9). Recent studies from our group and others have demonstrated that upregulating ABCA1 expression promotes cholesterol efflux and mitigates atherosclerosis (10–12), whereas suppressing ABCA1 reduces efflux and accelerates disease progression (13–15). These findings highlight ABCA1 as a pivotal therapeutic target for regulating macrophage cholesterol efflux and atherosclerosis. Therefore, identifying novel regulatory factors of ABCA1 provides crucial insights for preventing foam cell formation and alleviating atherosclerosis.

Gut microbiota dysbiosis and its metabolites influence the progression of atherosclerotic cardiovascular diseases (16). Trimethylamine-N-oxide (TMAO), short-chain fatty acids, phenylacetylglutamine (PAGln), and tryptophan metabolites participate in atherosclerosis pathogenesis by modulating macrophage differentiation and function (17, 18). Studies have indicated that the gut microbiota regulates RCT (19). 3-Hydroxystearic acid (C18-3OH), a recently identified gut microbiota-derived metabolite, has been linked to the increased abundance of Allobaculum, Holdemanella, and Parabacteroides in the gut microbiota (20). Previous research has demonstrated that C18-3OH levels impact host glucose, lipid, and energy metabolism (21). However, the relationship between C18-3OH and atherosclerotic cardiovascular disease remains unclear, and whether C18-3OH affects macrophage lipid accumulation is yet to be elucidated.

Paired box protein 8 (PAX-8), a transcription factor, regulates the expression of downstream target genes. PAX-8 is expressed in various human tissues and plays critical roles in tissue and organ development, cell proliferation, and apoptosis (22). Studies suggest that PAX-8 modulates angiogenesis and apoptosis, indicating its potential involvement in the regulation of atherosclerosis (23). However, the mechanistic role of PAX-8 in atherosclerosis remains unknown. Analysis via the PROMO database predicted multiple potential binding sites for PAX-8 within the ABCA1 promoter region.

N6-methyladenosine (m^6^A) modification is a predominant epigenetic modification in eukaryotic RNA. m^6^A plays a pivotal role in regulating mRNA splicing, stability, and protein translation (24). First, m^6^A modification can influence mRNA stability and thereby modulate gene expression. Second, it regulates mRNA translational efficiency, directly affecting protein synthesis. Additionally, m^6^A modification participates in chromatin remodeling by interacting with histone modifications and chromatin architecture, further regulating gene expression. The dynamic regulation of m^6^A is orchestrated by methyltransferases (“writers”), demethylases (“erasers”), and m^6^A-binding proteins (“readers”) (25). Recent studies have revealed that aberrant m^6^A modification contributes to the pathogenesis of atherosclerotic cardiovascular diseases (26). Predictive analysis using the SRAMP database (http://www.cuilab.cn/sramp) identified multiple high-confidence m^6^A modification sites on murine and human PAX-8 mRNA, suggesting that m^6^A may regulate PAX-8 expression.

Gut microbiota and its metabolites modulate the recruitment and activity of RNA m^6^A-modifying enzymes through epigenetic regulation in the host (27). α-Ketoglutarate-dependent dioxygenase AlkB homolog 5 (ALKBH5), a demethylase responsible for m^6^A modification, is widely expressed across tissues. ALKBH5 reverses mRNA m^6^A methylation via demethylation, thereby suppressing target gene expression (28, 29). Studies have indicated elevated ALKBH5 expression in cerebral infarction (30). Additionally, ALKBH5 participates in macrophage polarization and may be associated with acute myocardial infarction risk (31, 32). Atherosclerotic mice exhibit significantly elevated aortic m^6^A modification levels and markedly reduced ALKBH5 protein expression (33). These findings suggest a potential association between ALKBH5 and atherosclerosis pathogenesis.

This study first investigated the effects of C18-3OH on macrophage cholesterol efflux and ABCA1 expression at the cellular level, clarifying its role in mitigating lipid accumulation in macrophages. Combined with bioinformatics and high-throughput sequencing, molecular analyses revealed that C18-3OH suppressed ALKBH5, enhanced m^6^A modification of PAX-8 mRNA, upregulated PAX-8 expression, and subsequently increased ABCA1 levels, thereby promoting cholesterol efflux and reducing lipid deposition in foam cells. Further *in vivo* experiments elucidated the impact of C18-3OH on lipid metabolism and atherosclerosis progression, demonstrating the functional role of ALKBH5-mediated m^6^A modification in atherosclerotic pathogenesis and revealing the regulatory mechanism of ABCA1.

## 2 Materials and methods

### 2.1 Establishment of THP-1 macrophage-derived foam cells

THP-1 cells were subcultured using a semi-retention method. The culture medium consisted of RPMI 1640 supplemented with 10% fetal bovine serum (FBS) and was maintained in a humidified incubator at 37°C with 5% CO_2_. To differentiate THP-1 cells into macrophages, the culture medium was supplemented with 160 nmol/L phorbol 12-myristate 13-acetate (PMA) for 24 h. Subsequently, the cells were cultured in fresh medium for 6–12 h, followed by incubation with 50 μg/mL oxidized low-density lipoprotein (ox-LDL) for 48 h to induce foam cell formation.

### 2.2 CCK8 cell viability assay

THP-1 monocyte-derived foam cells were seeded into 96-well plates. Cells were treated with culture media containing C18-3OH at concentrations of 0, 25, 50, 100, 200, and 300 μM for 24 h to determine the optimal concentration. Based on preliminary results, 100 μM C18-3OH was selected for subsequent time-course experiments (0, 6, 12, 24, and 48 h of treatment). After incubation, 10 μL of CCK-8 reagent was added to each well and further incubated for 1 h. Optical density (OD) was measured at 450 nm using a microplate reader. Cell viability was calculated by normalizing the OD values of the treated groups to those of the untreated controls.

### 2.3 C18-3OH treatment

Foam cells were divided into control and C18-3OH-treated groups. The C18-3OH-treated group was incubated with 100 μM C18-3OH under standard culture conditions (37°C, 5% CO_2_) for 24 h. The control group received an equivalent volume of vehicle (1% DMSO) under identical incubation conditions. Subsequent experimental procedures were performed after treatment.

### 2.4 Microassay for intracellular cholesterol quantification

Isopropanol was used as the cholesterol extraction solvent. A 50 μmol/mL cholesterol standard solution was pre-diluted, and the working reagent was freshly prepared according to the manufacturer’s protocol. For every 5 million cells, 1 mL of extraction solvent was added. Cells were lysed via ice-bath ultrasonic disruption, followed by centrifugation at 10, 000 × g for 10 min in a pre-cooled (4°C) centrifuge. The supernatant was collected and stored on ice for further analysis. The working reagent was pre-warmed at 37°C for ≥10 min prior to use. The standard solution was serially diluted to 2.5, 1.25, 0.625, 0.3125, 0.15625, and 0 μmol/mL. In a 96-well plate, 20 μL of the standard solution and 180 μL of the working reagent were added to the standard wells, 20 μL of the extraction solvent and 180 μL of the working reagent were added to the blank wells, and 20 μL of the supernatant and 180 μL of the working reagent were added to the assay wells. After thorough mixing, the plate was incubated at 37°C in the dark for 30 min. Absorbance was measured at 500 nm using a microplate reader. A standard curve was plotted to calculate total cholesterol (TC) and free cholesterol (FC) levels. The cholesterol ester (CE) content was determined as CE = TC – FC.

### 2.5 Oil red O staining of cells

Cells were fixed with 4% paraformaldehyde for 15 min, followed by treatment with 60% isopropanol for 1 min. Freshly prepared Oil Red O working solution was used to stain the cells at room temperature for 25 min under light-protected conditions. The excess staining solution was removed by washing twice with phosphate-buffered saline (PBS). Nuclei were counterstained with hematoxylin working solution for 10 s, rinsed under running tap water for bluing, and finally mounted with glycerin for microscopic observation.

### 2.6 Quantitative real-time PCR

Total RNA was extracted by adding 1 mL of TRIzol reagent per well to completely lyse the cells. Subsequently, 200 μL of chloroform was added, mixed thoroughly, and centrifuged. The aqueous phase (approximately 200 μL) was transferred, mixed with 200 μL of isopropanol, and centrifuged to precipitate the RNA. The RNA pellet was washed with 75% ethanol prepared in DEPC-treated water, air-dried, and dissolved in 40 μL DEPC-treated water. RNA purity and concentration were determined using a NanoDrop spectrophotometer. cDNA was synthesized by reverse transcription using a thermal cycler. The primers were reconstituted, and quantitative PCR was performed using 2× SYBR Green qPCR Master Mix. Gene expression levels were calculated using the ΔΔCt method.

### 2.7 Protein extraction and quantification

Lysis buffer containing 1% protease inhibitor (PMSF) was added to each well of a 6-well plate (100–150 μL/well), followed by cell lysis on ice for 15 min. The cell suspension was collected and centrifuged to obtain a supernatant. Protein concentration was determined using the BCA assay, and the appropriate loading volume was calculated. The remaining protein samples in the Eppendorf tube were added to the corresponding buffer, denatured at 100°C for 5 min, and stored at −20°C for subsequent analyses.

### 2.8 Western blot analysis of protein expression

Separation gels with appropriate concentrations and 5% stacking gels were prepared based on the molecular weight of the target proteins. The electrophoresis chamber was assembled, and the comb was removed from the gel. The samples were carefully loaded into the wells using a pipette. Electrophoresis was performed at 80 V for 30 min at room temperature, followed by 120 V for 60 min. The gel was removed after electrophoresis. PVDF membranes were activated in absolute methanol for 1 min and equilibrated in the transfer buffer. The gel and membrane were sandwiched in a transfer cassette, immersed in transfer buffer, and subjected to wet transfer at 200 mA for 120 min. Post-transfer, membranes were blocked with 5% blocking buffer at room temperature for 1.5–2 h, then washed with TBST. Primary antibodies diluted in antibody dilution buffer were applied as follows: ABCA1 (Abcam, 1:1000), PAX-8 (Proteintech, 1:3000), ALKBH5 (Proteintech, 1:5000), ELAVL1 (Proteintech, 1:5000); GAPDH (Proteintech, 1:20, 000) and α-Tubulin (Proteintech, 1:20, 000) served as loading controls. The membranes were incubated with primary antibodies at 4°C overnight. After washing with TBST, the membranes were incubated with horseradish peroxidase (HRP)-conjugated secondary antibodies at room temperature for 60 min on a shaker. Unbound secondary antibodies were removed by washing with TBST. Protein bands were visualized using a chemiluminescence-imaging system.

### 2.9 Cholesterol efflux assay

THP-1 monocytes were differentiated into macrophages using PMA and cultured in serum-free medium for 6–12 h. Cells were labeled with 5 μg/mL NBD-cholesterol in RPMI 1640 medium containing 3% serum for 24 h, followed by three washes with PBS. The C18-3OH-treated group was incubated with 100 μM C18-3OH for 18 h, while the control group received an equivalent volume of the vehicle (1% DMSO). After treatment, the cells were incubated with apoA-I (20 μg/mL) for 6 h. The medium was discarded, and the cells were washed thrice with PBS. The final PBS wash was retained, and intracellular NBD-cholesterol fluorescence was visualized and captured using fluorescence microscopy.

For quantitative analysis, the cells were processed as described above. Following apoA-I incubation, the medium was collected, and cells were lysed with 0.1% Triton X-100 for 10 min after three PBS washes. The lysate was centrifuged, and the fluorescence intensities of both the medium and cell lysate were measured. Cholesterol efflux rate (%) was calculated as follows: Cholesterol efflux rate (%) = [Fluorescence of medium/(Fluorescence of medium + Fluorescence of lysate)] × 100%.

### 2.10 Bioinformatics analysis of PAX-8 binding sites

The PROMO online platform (https://alggen.lsi.upc.edu/recerca/menu_recerca.html) was used to predict the putative transcription factor binding sites within the DNA sequences. On the PROMO homepage, species and transcription factor information (PAX-8) were selected, and the promoter sequence of ABCA1 was submitted for analysis. The ABCA1 promoter sequence was retrieved from the NCBI database (https://www.ncbi.nlm.nih.gov/). The platform generated results including predicted binding motifs, confidence scores, and positional information for PAX-8 interaction with the promoter region.

### 2.11 Bioinformatics analysis of m^6^A modification sites on PAX-8 mRNA

The SRAMP database (http://www.cuilab.cn/sramp) was used to predict m^6^A modification sites on mammalian RNA sequences. The mRNA sequence of the target gene PAX-8 was retrieved from the NCBI database (https://www.ncbi.nlm.nih.gov/). This sequence was pasted into the “FASTA genomic sequence” field on the SRAMP prediction page. Appropriate analysis parameters were selected, and the “Submit” button was clicked to generate the predicted m^6^A modification sites, including their positional coordinates and confidence scores.

### 2.12 Bioinformatics analysis of proteins regulating PAX-8 methylation in THP-1 cells

The RM2Target database (http://rm2target.canceromics.org/#/home) is a comprehensive platform for predicting RNA modification-associated regulatory proteins and their target genes. On the RM2Target homepage, users can input parameters such as the Weighted Enrichment Ratio (WER), target gene (PAX-8), or species information to retrieve candidate proteins involved in regulating PAX-8 methylation modifications within the specified cell line (e.g., THP-1) or tissue type.

### 2.13 Detection of m^6^A modification levels on PAX-8 mRNA

The m^6^A modification levels of PAX-8 mRNA were assessed using the Magna MeRIP m^6^A Kit. Total RNA was isolated using TRIzol reagent, followed by ribosomal RNA depletion and fragmentation. RNA fragments were incubated with magnetic beads conjugated to an m^6^A-specific antibody to enrich the methylated RNA. After elution and purification, m^6^A-modified PAX-8 mRNA was quantified using RT-qPCR with sequence-specific primers.

### 2.14 siRNA transfection for target gene knockdown

Foam cells were transfected with pre-designed siRNAs. PAX-8 siRNA and negative control siRNA (siNC) were synthesized by RiboBio (Guangzhou, China). Transfection was performed according to the manufacturer’s instructions by adding the appropriate reagents and viral vectors. The experimental groups were divided as follows: DMSO + siNC, DMSO + siPAX-8, C18-3OH + siNC, and C18-3OH + siPAX-8. Each group was set up in triplicate. Subsequent experiments were conducted after transfection. The PAX-8 siRNA sequences were Sense: 5’-CAGGAUAGCU GCCGACUAAdTdT-3’ and Antisense: 5’-UUAGUCGGCAGCUAUCCUGdTdT-3’.

### 2.15 Lentiviral transfection for overexpression of target genes

Foam cells were transfected with pre-packaged lentiviral vectors. Lentiviruses carrying PAX-8 (LV-PAX-8) and ALKBH5 (LV-ALKBH5) were constructed by Genepharma (Shanghai, China). Transfection was performed according to the manufacturer’s protocol, with appropriate reagents and viral particles. Each experimental group was analyzed in triplicate. Subsequent analyses were performed after transfection. The human PAX-8 (NM_003466.4) and ALKBH5 (NM_0117758.4) transcripts were cloned from the Genepharma cDNA library.

### 2.16 ChIP-seq analysis for transcription factor target genes

Following C18-3OH treatment, the cells were washed to remove the culture medium and stored at −80°C for subsequent analysis. Frozen cell pellets were thawed and subjected to chromatin immunoprecipitation sequencing (ChIP-Seq) to identify PAX-8 target genes. Cell samples were processed through lysis, sonication, immunoprecipitation, and cross-linking to obtain DNA. The purified DNA fragments, after precipitation and dilution, were used to construct a ChIP-seq library for sequencing. The ChIP-seq experiment was independently repeated once (34). The experimental procedures were performed by Shanghai Kangcheng Biotechnology Co., Ltd., with standardized protocols executed by trained personnel. Data visualization, including the genomic localization of target genes, was conducted using the UCSC Genome Browser (http://genome.ucsc.edu/).

### 2.17 ChIP-qPCR validation of PAX-8 binding to target genes

ChIP-derived samples were eluted and purified to obtain the DNA fragments. The control groups consisted of non-enriched input samples without immunoprecipitation (IP). ChIP-qPCR was performed to validate the binding of PAX-8 to the ABCA1. Primers targeting the ABCA1-specific binding sites identified by ChIP-Seq were designed using Primer Primer 5.0 software.

### 2.18 Husbandry of apoE^−/−^ mice and untargeted serum metabolomics via UHPLC-OE-MS

Male apoE^−/−^ mice aged 6–8 weeks were housed in the Experimental Animal Center of the University of South China, China. Mice were individually housed with unrestricted access to water and standard chow, under controlled conditions (20 ± 2°C, 50–60% humidity, 12-hour light/dark cycle). After a 1-week acclimatization period, the mice were randomly divided into two groups (n = 6/group): control group (standard diet) and high-fat diet (HFD) group (fed with HFD). All procedures were approved by the Institutional Animal Care and Use Committee of the University of South China.

After 12 weeks, the mice were anesthetized via intraperitoneal injection of 20% urethane (5 mL/kg). Blood was collected in EDTA-coated tubes and allowed to stand at room temperature. Cardiac perfusion with PBS was performed to remove residual blood from tissues. The aortas were dissected to evaluate atherosclerotic plaque formation, confirming the successful establishment of the HFD-induced atherosclerosis model. Serum was isolated by centrifuging clotted blood and stored at −80°C.

Serum samples were subjected to untargeted metabolomic profiling using ultra-high-performance liquid chromatography coupled with orbitrap mass spectrometry (UHPLC-OE-MS) to analyze alteractions in fatty acid metabolites. Mass spectrometry can be utilized for quantitative analysis of samples (35). The experimental procedures were performed by trained personnel at Jiangsu BioTREE Biotechnology Co., Ltd.

### 2.19 Husbandry and modeling of apoE^−/−^ mice

A total of forty-eight 6–8-week-old male apoE^−/−^ mice were housed in the Experimental Animal Center of the University of South China under standardized conditions (as described previously). The mice were randomly divided into four groups (n=12/group): control, C18-3OH, C18-3OH+AAV-NC, and C18-3OH+AAV-ALKBH5. All mice were fed a high-fat diet (HFD) for 12 weeks. Body weights were monitored weekly. The C18-3OH, C18-3OH+AAV-NC, and C18-3OH+AAV-ALKBH5 groups received daily intraperitoneal injections of 100 μM C18-3OH (100 μL/day; vehicle: 1% DMSO + 99% PBS), while the control group was administered an equivalent volume of vehicle. On day 0, the C18-3OH+AAV-ALKBH5 group underwent tail vein injection of adeno-associated virus (AAV) overexpressing ALKBH5, and the C18-3OH+AAV-NC group received a negative control AAV. All protocols were approved by the Institutional Animal Care and Use Committee of the University of South China, China.

### 2.20 Serum and tissue specimen collection

After 12 weeks, the mice were euthanized for subsequent procedures. Blood was collected in microcentrifuge tubes and allowed to clot at room temperature. Following fixation, the heart was exposed, and systemic perfusion with PBS was performed to remove residual blood from tissues. The surrounding tissues were dissected to observe aortic plaque formation. The aortas and hearts were isolated, and six vessels per group were preserved for protein and mRNA quantification. The remaining cardiac and aortic tissues were fixed in 4% paraformaldehyde for histological sectioning and staining. Clotted blood was centrifuged after 4 h of room temperature incubation to isolate serum, which was stored at −80°C for further analysis.

### 2.21 Serum biochemical parameter analysis

Serum alanine aminotransferase (ALT) and aspartate aminotransferase (AST) activities, serum creatinine (Scr), blood urea nitrogen (BUN), total cholesterol (TC), low-density lipoprotein cholesterol (LDL-C), high-density lipoprotein cholesterol (HDL-C), and triglycerides (TG) levels were measured using commercially available assay kits according to the manufacturer’s protocols. All measurements were performed in technical replicates to minimize the variability of the experiment.

### 2.22 Aortic oil red O staining

Aortas were dissected from mice and fixed in 4% paraformaldehyde for 24 h, followed by PBS washes. The perivascular adipose tissue was carefully removed, and small vascular branches were trimmed. The aortas were longitudinally incised using Vannas scissors, including the aortic arch trifurcation and iliac artery branches. The Oil Red O working solution was prepared by mixing Oil Red O stock solution with double-distilled water (6:4, v/v), filtered under light-protected conditions, and aliquoted into centrifuge tubes. The incised aortas were immersed in Oil Red O working solution for 20 min and then rinsed sequentially in 75% ethanol (3 s) and PBS (10 s). The aortas were flattened on adhesive-coated slides with a scale bar positioned alongside, and images were captured under a microscope for plaque quantification.

### 2.23 Hematoxylin and eosin staining of aortic sinus

Mouse hearts were embedded in OCT compound and subjected to serial cryosectioning of the aortic sinus at 7 μm thickness. Tissue sections were fixed with 4% paraformaldehyde for 15 min, followed by three washes with PBS. The sections were stained with hematoxylin for 5 min, rinsed to remove excess dye, and differentiated for 30 s. Bluing was achieved by rinsing under running tap water for 10 min. Counterstaining was performed with eosin solution for 1 min, followed by a 5-minute rinse with running water. The sections were then dehydrated through a graded ethanol series, cleared in xylene, and mounted with neutral resin for microscopic examination.

### 2.24 Oil red O staining of aortic sinus

Tissue sections were fixed with 4% paraformaldehyde for 15 min and washed three times with PBS. The sections were immersed in 60% isopropanol for 5 s, blotted dry, and stained with Oil Red O working solution for 20 min. Excess dye was removed by differentiation in 60% isopropanol for 5 s to clarify the interstitial matrix, followed by three washes with running water. The slides were mounted with glycerol gelatin and imaged using a microscope-equipped digital capture system.

### 2.25 Masson’s trichrome staining of aortic sinus

Tissue sections were fixed with 4% paraformaldehyde for 15 min and washed three times with PBS. The sections were stained with hematoxylin for 5 min, rinsed to remove excess dye, and blued under running tap water. Subsequently, the sections were stained with Ponceau S and Acid Fuchsin solution for 5 min, followed by Aniline Blue solution for 2 min. Excess dye was removed by rinsing with a weak acid working solution for 1 min. Finally, the sections were dehydrated through a graded ethanol series, cleared in xylene, and mounted with neutral resin for histological analysis.

### 2.26 Data analysis and processing

Statistical analyses were performed using SPSS 26.0, and graphs were generated using GraphPad Prism 8.0. Data normality was assessed using the *Shapiro-Wilk test*, and homogeneity of variances was evaluated using *Levene’s test*. Comparisons between two groups were analyzed using *Student’s t-test* (for equal variances). Comparisons among multiple groups were assessed using one-way ANOVA followed by *Tukey’s post hoc test* (for equal variances). If the assumption of equal variances was violated, *Welch’s t-test* or *Welch’s* ANOVA was applied. All experiments were performed at least three times. *In vitro* data are presented as mean ± standard deviation (SD), and *in vivo* results are expressed as mean ± standard error of the mean (SEM). A significance threshold of *P* < 0.05 was applied to denote statistical significance.

## 3 Results

### 3.1 C18-3OH reduces cholesterol content in foam cells and inhibits intracellular lipid accumulation

**Figure 1:**
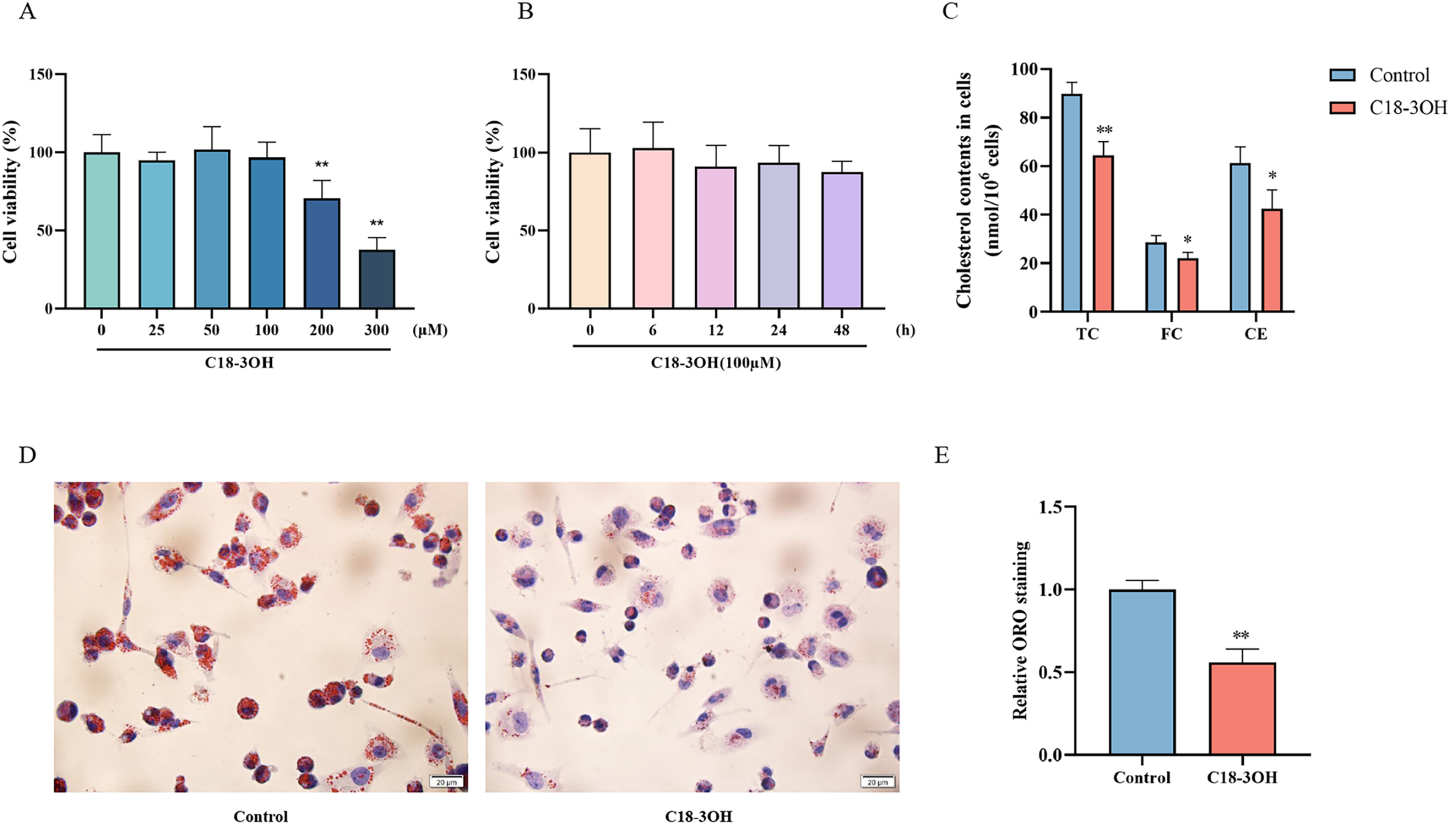
Effects of C18-3OH on cholesterol content and intracellular lipid accumulation in foam cells. **(A)** Effect of C18-3OH at varying concentrations (0, 25, 50, 100, 200, and 300 μM) on viability of foam cells after 24 h treatment. ^**^P < 0.01 vs. 0 μM group, n = 5. **(B)** Time-dependent effect of 100 μM C18-3OH on viability of foam cells. n = 5. **(C)** C18-3OH reduces intracellular cholesterol content in foam cells. **(D)** Oil Red O staining of lipid droplets in foam cells treated with C18-3OH; scale bar = 20 μm. **(E)** Quantitative analysis of Oil Red O-stained lipid area. *P < 0.05, ^**^P < 0.01 vs. control, n = 3.

Macrophage lipid accumulation is a critical pathological event that drives foam cell formation and atherosclerosis. To investigate whether C18-3OH modulates lipid accumulation in foam cells, the optimal treatment concentration and duration were first determined. Foam cells were treated with C18-3OH at varying concentrations (0, 25, 50, 100, 200, 300 μM) for 24 h, and 100 μM was selected as the optimal concentration. Time-dependent effects (0, 6, 12, 24, 48 h) on cell viability were assessed via the CCK-8 assay. Results indicated reduced cell viability at concentrations exceeding 200 μM, while 100 μM C18-3OH showed no significant impact on viability within 48 h (**Figures 1A, B**). Based on these findings, subsequent experiments utilized 100 μM C18-3OH with a 24 h treatment period.

For experimental groups, foam cells treated with 100 μM C18-3OH for 24 h were analyzed for intracellular lipid content using biochemical assays and Oil Red O staining. Compared to controls, C18-3OH-treated cells exhibited significant reductions in cholesterol esters, total cholesterol, and free cholesterol levels (**Figure 1C**). Furthermore, lipid droplet formation was markedly diminished in the C18-3OH group (**Figures 1D, E**). These results demonstrate that C18-3OH effectively reduces cholesterol levels and suppresses lipid accumulation in foam cells.

### 3.2 C18-3OH upregulates ABCA1 expression in foam cells and promotes cholesterol efflux

**Figure 2:**
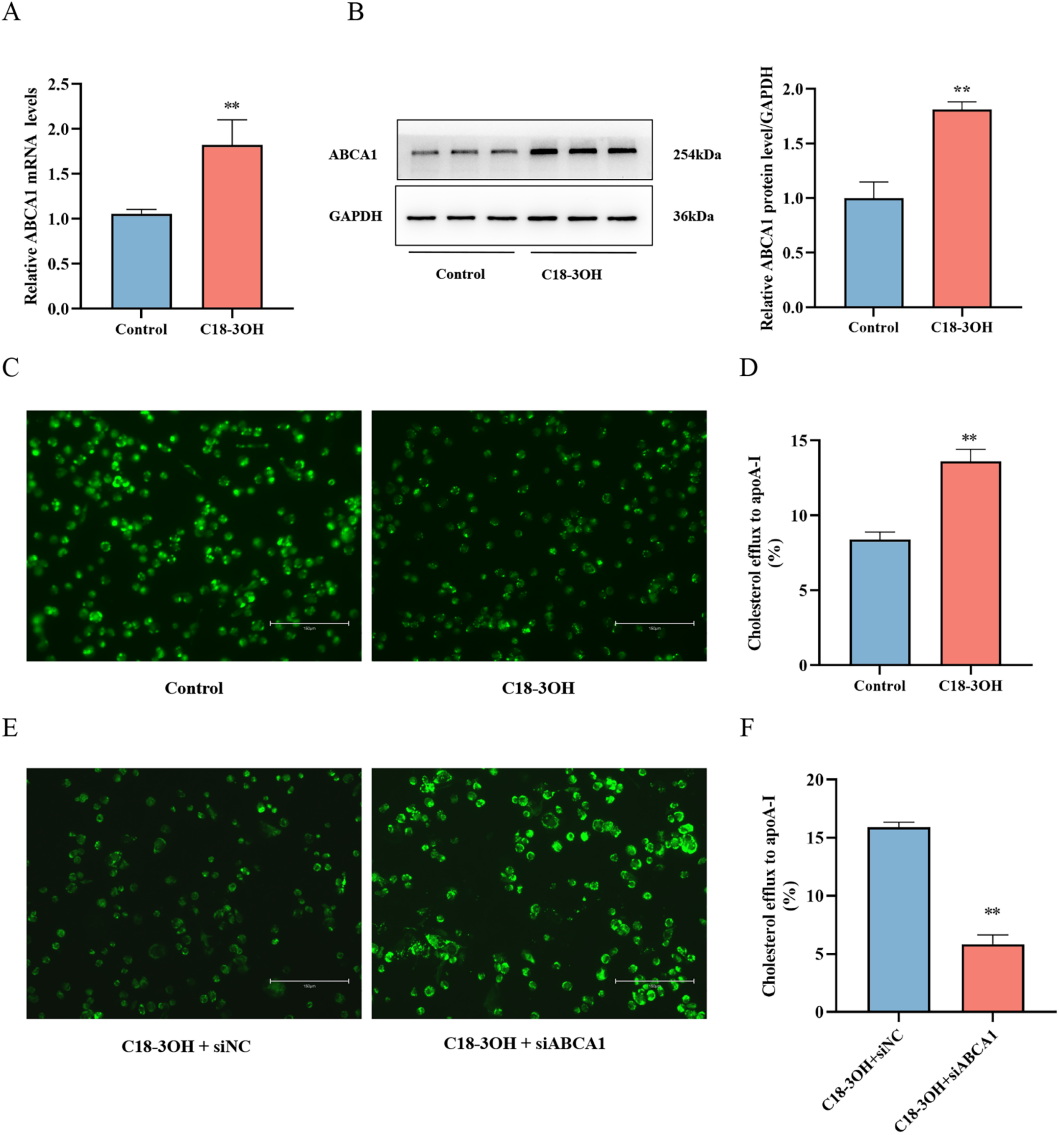
Effects of C18-3OH on ABCA1 expression and cholesterol efflux in foam cells. **(A)** RT-qPCR analysis of ABCA1 mRNA expression in foam cells treated with C18-3OH. **(B)** Western blot analysis of ABCA1 protein levels in foam cells treated with C18-3OH. **(C)** NBD-cholesterol fluorescence intensity in foam cells; scale bar = 150 μm. **(D)** Effect of C18-3OH on cholesterol efflux efficiency. ^**^P < 0.01 vs. control, n = 3. **(E)** NBD-cholesterol fluorescence intensity in ABCA1-knockdown foam cells; scale bar = 150 μm. **(F)** Effect of C18-3OH on cholesterol efflux efficiency in ABCA1-knockdown foam cells. ^**^P < 0.01 vs. C18-3OH+siNC, n = 3.

ABCA1, a critical membrane protein that mediates intracellular cholesterol efflux, plays a pivotal role in reducing lipid accumulation in foam cells. To explore the underlying mechanism, we first examined the effects of C18-3OH on cholesterol efflux-related protein expression. Foam cells treated with 100 μM C18-3OH for 24 h were subjected to RT-qPCR and Western blot analyses to assess ABCA1 expression. The results showed that both mRNA and protein levels of ABCA1 were significantly elevated in the C18-3OH group compared to the control group (**Figures 2A, B**).

ApoA-I serves as a receptor for extracellular cholesterol uptake. To elucidate the impact of C18-3OH on cholesterol efflux, we measured cholesterol efflux levels using NBD-cholesterol-labeled macrophages. The C18-3OH group exhibited markedly enhanced cholesterol efflux to apoA-I compared to the control group (**Figures 2C, D**). In our study, we used siRNA to knock down ABCA1 expression in foam cells. The results showed that in ABCA1-knockdown cells, the promoting effect of C18-3OH on cholesterol efflux was significantly attenuated. This data confirms that the upregulation of ABCA1 is essential for C18-3OH-mediated promotion of cholesterol efflux (**Figures 2E, F**). These findings suggest that C18-3OH upregulates ABCA1 expression in THP-1 macrophage-derived foam cells, thereby promoting cholesterol efflux.

### 3.3 C18-3OH promotes ABCA1 expression and cholesterol efflux in foam cells via PAX-8 upregulation

**Figure 3:**
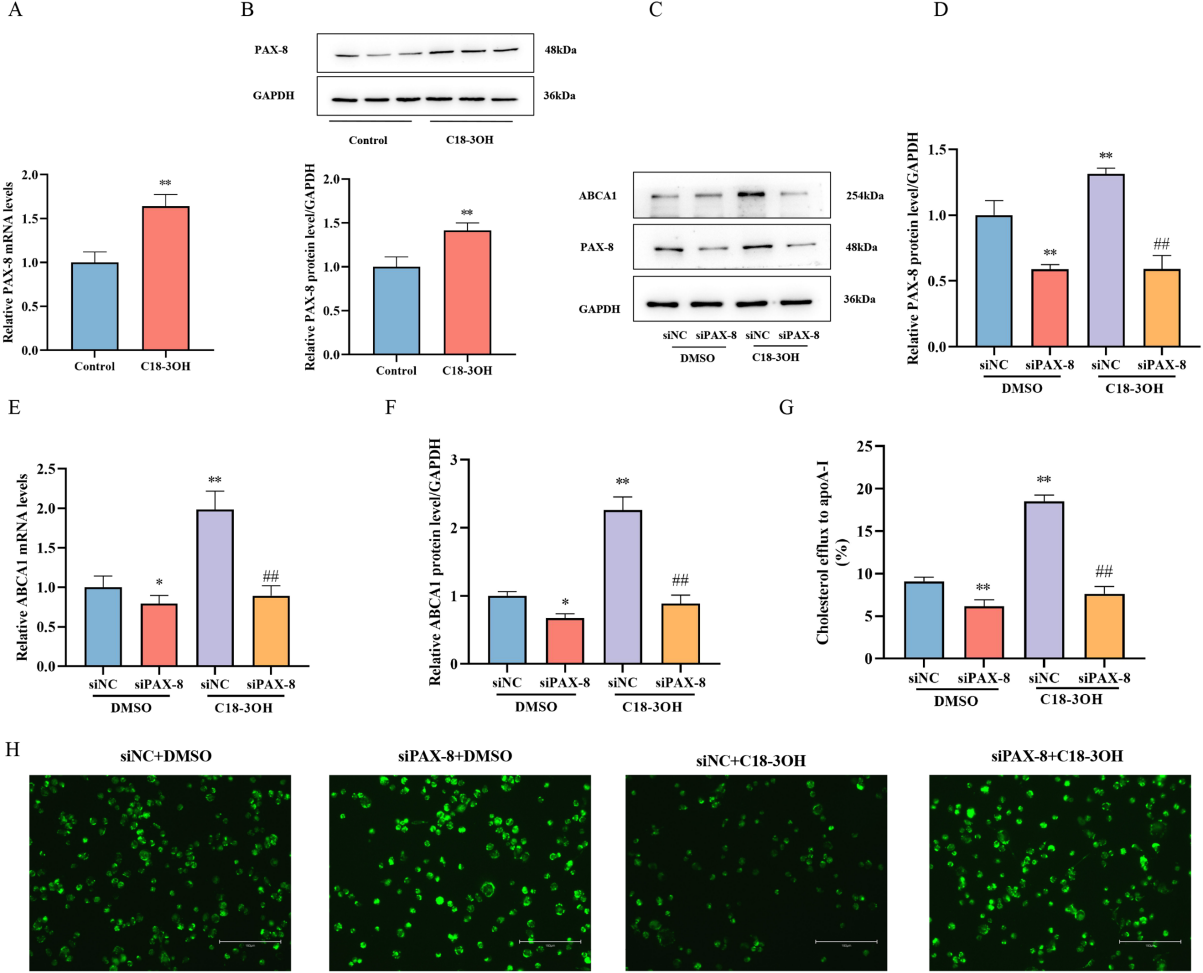
C18-3OH modulates ABCA1 expression and cholesterol efflux in foam cells via PAX-8 upregulation. **(A)** RT-qPCR analysis of PAX-8 mRNA expression in foam cells treated with C18-3OH. **(B)** Western blot analysis of PAX-8 protein expression in foam cells treated with C18-3OH. ^**^P < 0.01 vs. control, n = 3. **(C)** Western blot detection of PAX-8 and ABCA1 protein expression in foam cells following PAX-8 knockdown. **(D)** Quantitative analysis of PAX-8 protein expression (Panel C). **(E)** RT-qPCR analysis of ABCA1 mRNA expression in foam cells after PAX-8 knockdown. **(F)** Quantitative analysis of ABCA1 protein expression (Panel C). **(G)** Effects of C18-3OH and siPAX-8 on cholesterol efflux in foam cells. ^*^P < 0.05, ^**^P < 0.01 vs. siNC + DMSO; ^##^*P* < 0.01 vs. siNC + C18-3OH, n = 3. **(H)** NBD-cholesterol fluorescence intensity in foam cells; scale bar = 150 μm.

PAX-8, a key transcription factor of the PAX family, has not been fully characterized in foam cell formation and atherosclerosis. To investigate whether C18-3OH modulates PAX-8 expression, foam cells were treated with 100 μM C18-3OH for 24 h (control groups received equivalent volume of DMSO), followed by RT-qPCR and Western blot analyses. The results demonstrated a significant upregulation of PAX-8 mRNA and protein levels in the C18-3OH group compared to the controls (**Figures 3A, B**). To determine the role of PAX-8 in C18-3OH-mediated regulation of ABCA1 expression, PAX-8 siRNA was transfected into foam cells to knock down PAX-8. RT-qPCR and Western blotting revealed that PAX-8 siRNA transfection markedly reduced PAX-8 expression and downregulated ABCA1 mRNA and protein levels. Notably, in C18-3OH-treated cells, ABCA1 expression was significantly attenuated in the siPAX-8 + C18-3OH group compared with that in the siNC + C18-3OH group (**Figures 3C, F**). These findings indicate that C18-3OH enhances ABCA1 expression in foam cells via PAX-8 upregulation.

Cholesterol efflux assays were performed to assess whether C18-3OH modulates cholesterol efflux via PAX-8. PAX-8 siRNA-transfected foam cells exhibited higher intracellular NBD-cholesterol fluorescence intensity and lower efflux efficiency to apoA-I compared to controls. While C18-3OH treatment alone reduced intracellular fluorescence and enhanced efflux efficiency, this effect was partially reversed in the siPAX-8 + C18-3OH group (**Figures 3G, H**). Collectively, these results demonstrate that C18-3OH promotes cholesterol efflux to ApoA-I by upregulating PAX-8 expression, and that PAX-8 silencing partially abolishes this regulatory effect.

### 3.4 PAX-8 binds to ABCA1 and regulates its expression

**Figure 4:**
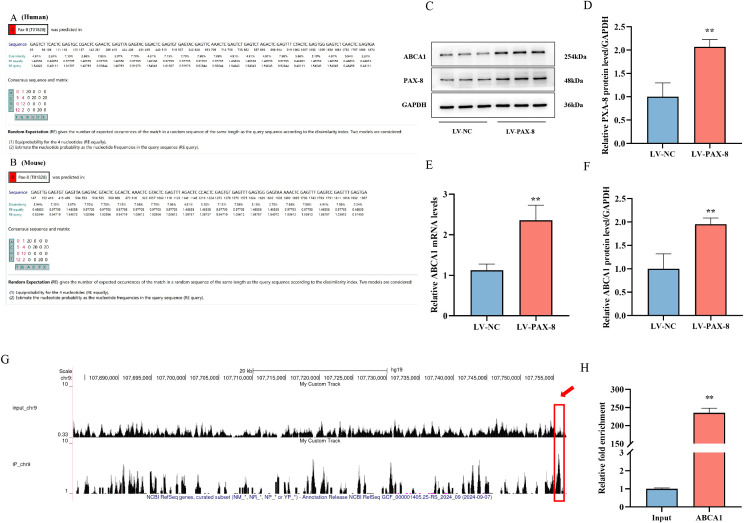
PAX-8 binds to and regulates ABCA1 expression. **(A)** Predicted binding sites between PAX-8 and ABCA1 in humans. **(B)** Predicted binding sites between PAX-8 and ABCA1 in mice. **(C)** Western blot analysis of PAX-8 and ABCA1 protein expression in foam cells following PAX-8 overexpression. **(D)** Quantitative analysis of PAX-8 protein expression (Panel C). **(E)** RT-qPCR analysis of ABCA1 mRNA expression in foam cells after PAX-8 overexpression. **(F)** Quantitative analysis of ABCA1 protein expression (Panel C). ^**^P < 0.01 vs. LV-NC, n = 3. **(G)** ChIP-Seq identification of PAX-8 binding to the ABCA1 locus. Genomic tracks showing PAX-8 binding to the ABCA1 locus in the Input (negative control, top) and ChIP (PAX-8-enriched, bottom) groups. The y-axis indicates relative enrichment of ChIP-Seq signals. The x-axis represents genomic coordinates. **(H)** ChIP-qPCR validation of PAX-8 targeting regulation of ABCA1. ^**^P < 0.01 vs. Input, n = 3.

Previous studies have confirmed that PAX-8 regulates ABCA1 expression. To elucidate the underlying mechanism, PAX-8 binding sites were predicted using the PROMO online database (https://alggen.lsi.upc.edu/recerca/menu_recerca.html). Analysis revealed multiple potential PAX-8 binding sites within the ABCA1 promoter regions in both human and murine genomes (**Figures 4A, B**). To validate whether PAX-8 modulates ABCA1 expression in foam cells, PAX-8-overexpressing lentivirus was transfected into foam cells (the control groups received negative control virus). RT-qPCR and Western blot analyses demonstrated that PAX-8 overexpression significantly upregulated both PAX-8 and ABCA1 mRNA and protein levels compared to controls (**Figures 4C–F**), indicating that PAX-8 promotes ABCA1 expression in THP-1 macrophage-derived foam cells.

To determine the direct binding between PAX-8 and ABCA1, ChIP-Seq was performed on C18-3OH-treated foam cells. Sequencing identified a PAX-8 binding site within the ABCA1 gene locus (chr9:107755317-107755882), which was visualized using the UCSC Genome Browser (http://genome.ucsc.edu/). The detailed bioinformatics analysis of the ChIP-Seq data identified significant peaks within the intergenic region proximal to the ABCA1 transcription start site. (**Figure 4G**). For validation, primers targeting this region were designed, and ChIP-qPCR was used to confirm PAX-8 enrichment at the ABCA1 locus. The results showed a significant upregulation of ABCA1 mRNA in PAX-8-enriched samples compared to the input controls (**Figure 4H**). These data conclusively demonstrate that PAX-8 binds to ABCA1 and regulates its expression at the transcriptional level.

### 3.5 C18-3OH enhances m^6^A methylation of PAX-8 mRNA in foam cells

**Figure 5:**
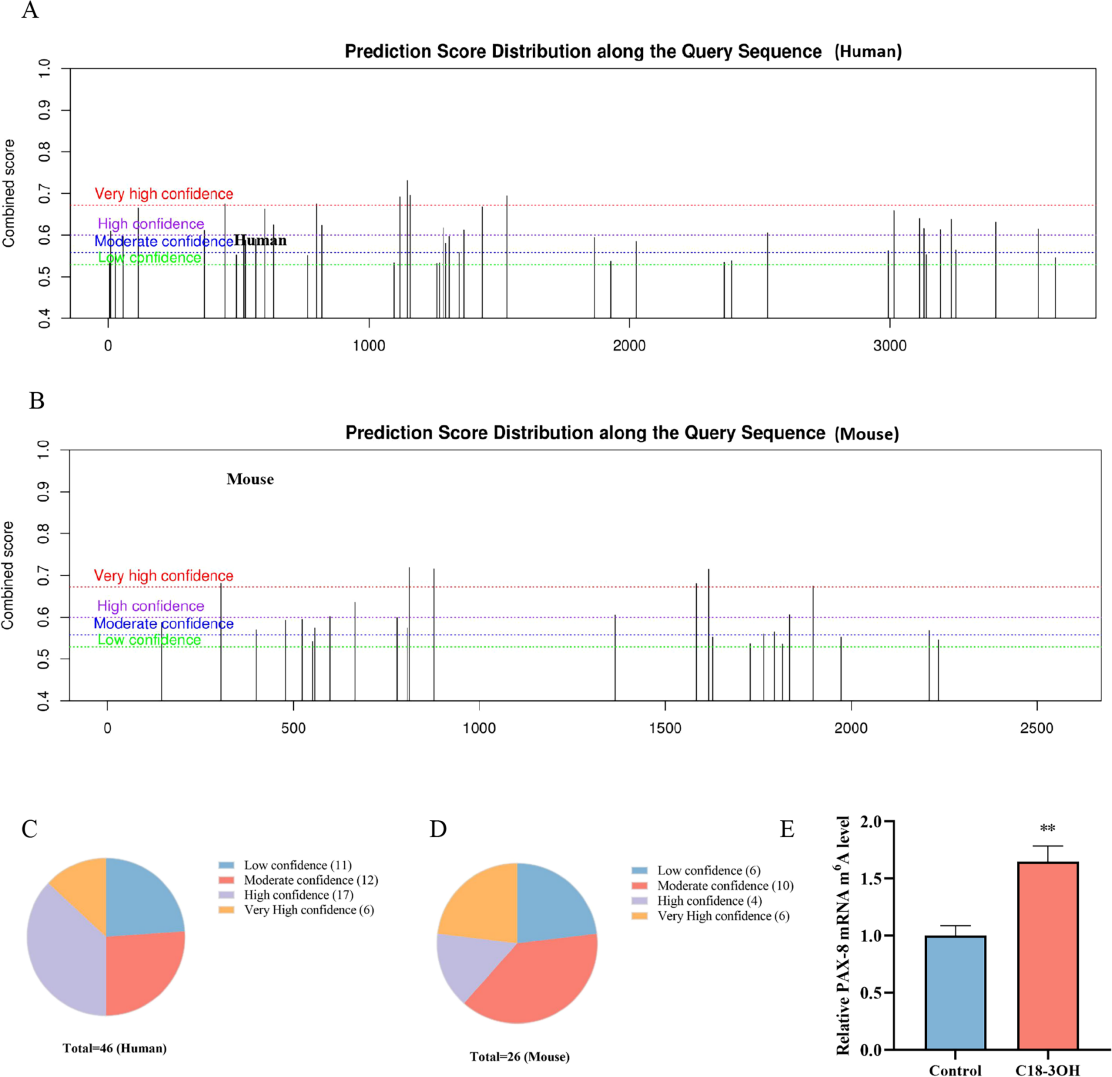
Effects of C18-3OH on m^6^A modification of PAX-8 mRNA in foam cells. **(A)** Predicted score distribution of the human PAX-8 gene query sequence. **(B)** Predicted score distribution of the murine PAX-8 gene query sequence. **(C)** Partitioned statistical plot of predicted scores for the human PAX-8 gene query sequence. **(D)** Partitioned statistical plot of predicted scores for the murine PAX-8 gene query sequence. **(E)** MeRIP-qPCR analysis of the effect of C18-3OH on m^6^A modification of PAX-8 mRNA in foam cells. ^**^P < 0.01 vs. control, n = 3.

m^6^A modification is a pivotal epitranscriptomic mechanism in eukaryotic mRNA. To explore whether the regulation of PAX-8 expression by C18-3OH is through the m^6^A modification pathway, PAX-8 mRNA m^6^A modification sites were predicted using the SRAMP online platform (http://www.cuilab.cn/sramp). Analysis identified 23 high-confidence m^6^A sites in human PAX-8 mRNA and 10 high-confidence sites in murine PAX-8 mRNA (**Figures 5A–D**), providing molecular evidence for PAX-8 m^6^A methylation. MeRIP-qPCR was performed to quantify PAX-8 mRNA m^6^A levels in foam cells. Compared to controls, C18-3OH treatment significantly increased PAX-8 mRNA m^6^A methylation (**Figure 5E**), indicating that C18-3OH enhances m^6^A modification of PAX-8 mRNA in THP-1 macrophage-derived foam cells.

### 3.6 C18-3OH suppresses ALKBH5 expression in foam cells

**Figure 6:**
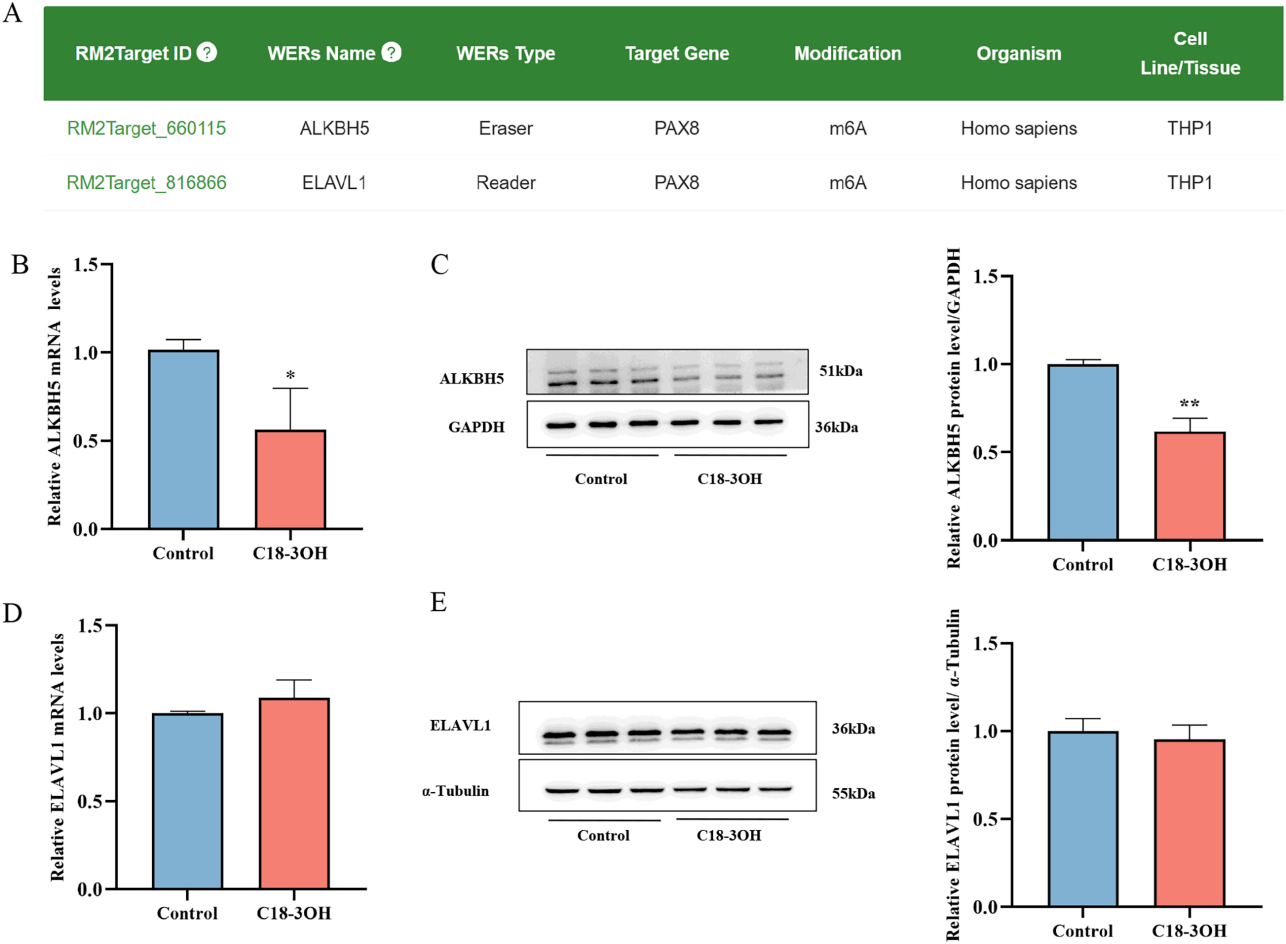
Effects of C18-3OH on ALKBH5 expression in foam cells. **(A)** Bioinformatic analysis of methylation-associated proteins regulating PAX-8 in THP-1 cells. **(B)** RT-qPCR analysis of ALKBH5 mRNA expression in foam cells treated with C18-3OH. **(C)** Western blot analysis of ALKBH5 protein expression in foam cells treated with C18-3OH. **(D)** RT-qPCR analysis of ELAVL1 mRNA expression in foam cells treated with C18-3OH. **(E)** Western blot analysis of ELAVL1 protein expression in foam cells treated with C18-3OH. ^*^P < 0.05, ^**^P < 0.01 vs. control, n = 3.

To explore the mechanism underlying C18-3OH-mediated m^6^A methylation of PAX-8 mRNA, potential regulators of PAX-8 mRNA m^6^A modification in THP-1 cells were predicted using the RM2Target database (http://rm2target.canceromics.org/#/home). Results suggested that PAX-8 m^6^A methylation in THP-1 cells may be regulated by the demethylase ALKBH5 and the reader protein ELAVL1 (**Figure 6A**). ALKBH5 and ELAVL1 expression levels in foam cells were analyzed by RT-qPCR and Western blotting. Compared to controls, C18-3OH-treated cells exhibited significant downregulation of ALKBH5 mRNA and protein expression (**Figures 6B, C**), whereas ELAVL1 mRNA and protein levels remained unaffected (**Figures 6D, E**). These findings indicate that C18-3OH specifically inhibits the expression of the m^6^A demethylase ALKBH5 but does not alter the expression of the reader protein ELAVL1.

### 3.7 ALKBH5 mediates regulatory effects of C18-3OH on PAX-8/ABCA1 expression, PAX-8 mRNA m^6^A methylation, and cholesterol efflux

**Figure 7:**
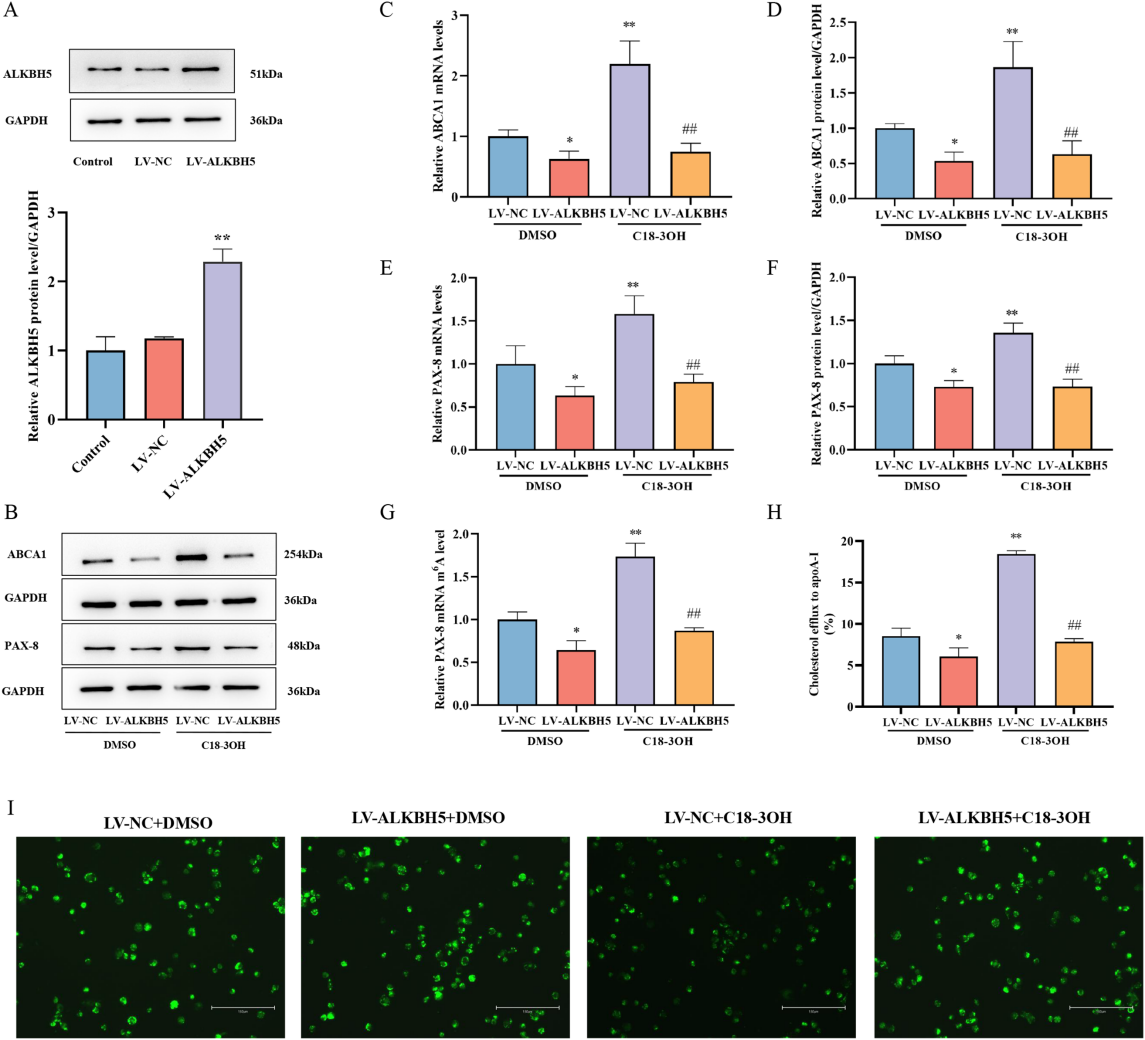
ALKBH5 mediates C18-3OH regulation of PAX-8 and ABCA1 expression, PAX-8 mRNA m^6^A modification, and cholesterol efflux. **(A)** Western blot analysis of ALKBH5 expression in foam cells following lentiviral transfection for ALKBH5 overexpression. ^**^P < 0.01 vs. control, n = 3. **(B)** Western blot analysis of PAX-8 and ABCA1 protein expression in foam cells following ALKBH5 overexpression. **(C)** RT-qPCR analysis of ABCA1 mRNA expression in foam cells after ALKBH5 overexpression. **(D)** Quantitative analysis of ABCA1 protein expression (Panel B). **(E)** RT-qPCR analysis of PAX-8 mRNA expression in foam cells after ALKBH5 overexpression. **(F)** Quantitative analysis of PAX-8 protein expression (Panel B). **(G)** MeRIP-qPCR analysis of PAX-8 mRNA m6A modification in foam cells following ALKBH5 overexpression. **(H)** Effect of C18-3OH and LV-ALKBH5 on cholesterol efflux in foam cells. **(I)** NBD-cholesterol fluorescence intensity in foam cells; scale bar = 150 μm. ^*^P < 0.05, ^**^P < 0.01 vs. LV-NC + DMSO; ^##^*P* < 0.01 vs. LV-NC + C18-3OH, n = 3.

To investigate the role of ALKBH5 in C18-3OH-mediated regulation of PAX-8 and ABCA1, ALKBH5 overexpression was first validated. Foam cells transfected for 24 h with LV-NC (negative control) or LV-ALKBH5 were analyzed by Western blotting. LV-ALKBH5 significantly increased ALKBH5 protein levels compared to LV-NC and untreated controls, confirming successful overexpression (**Figure 7A**). Foam cells transfected with LV-ALKBH5 or LV-NC were treated with 100 μM C18-3OH for 24 h. RT-qPCR and Western blot revealed that LV-ALKBH5 + DMSO reduced PAX-8 and ABCA1 mRNA/protein levels compared to LV-NC + DMSO, while LV-NC + C18-3OH markedly upregulated their expression. Notably, LV-ALKBH5 + C18-3OH attenuated the C18-3OH-induced upregulation of PAX-8 and ABCA1 (**Figures 7B–F**), demonstrating that ALKBH5 overexpression suppresses the effects of C18-3OH on PAX-8/ABCA1 expression.

To determine whether C18-3OH modulates PAX-8 mRNA m^6^A levels via ALKBH5, foam cells transfected for 24 h with LV-ALKBH5 or LV-NC were treated with C18-3OH for 24 h, followed by MeRIP-qPCR. Compared to the LV-NC + DMSO group, the LV-ALKBH5 + DMSO group exhibited reduced PAX-8 mRNA m^6^A methylation, while the LV-NC + C18-3OH group showed increased methylation. Furthermore, the LV-ALKBH5 + C18-3OH group displayed significantly diminished m^6^A modification compared to that of the LV-NC + C18-3OH group (**Figure 7G**), indicating that overexpression of ALKBH5 suppresses C18-3OH-induced enhancement of PAX-8 mRNA m^6^A methylation.

Finally, cholesterol efflux was assessed. The LV-ALKBH5 + DMSO group exhibited elevated intracellular NBD-cholesterol fluorescence intensity (indicating impaired efflux) compared to LV-NC + DMSO controls, whereas the LV-NC + C18-3OH group showed reduced fluorescence (enhanced efflux). Notably, the LV-ALKBH5 + C18-3OH group reversed the C18-3OH-mediated improvement in cholesterol efflux to apoA-I (**Figures 7H, I**). These results demonstrate that ALKBH5 overexpression inhibits the promotive effects of C18-3OH on cholesterol efflux.

To validate whether ALKBH5 affects cholesterol efflux in foam cells, intracellular fluorescence intensity and cholesterol efflux efficiency to apoA-I were measured. Compared to the LV-NC + DMSO group, the LV-ALKBH5 + DMSO group exhibited increased intracellular cholesterol fluorescence intensity and reduced efflux efficiency. Conversely, the LV-NC + C18-3OH group showed decreased fluorescence intensity and improved efflux efficiency. Notably, the LV-ALKBH5 + C18-3OH group reversed these effects, displaying elevated fluorescence intensity and reduced efflux efficiency compared to the LV-NC + C18-3OH group (**Figures 7H, I**). These results demonstrate that ALKBH5 overexpression suppresses C18-3OH-mediated promotion of cholesterol efflux in foam cells.

### 3.8 Reduced serum C18-3OH levels in apoE^-/-^ mice

**Figure 8:**
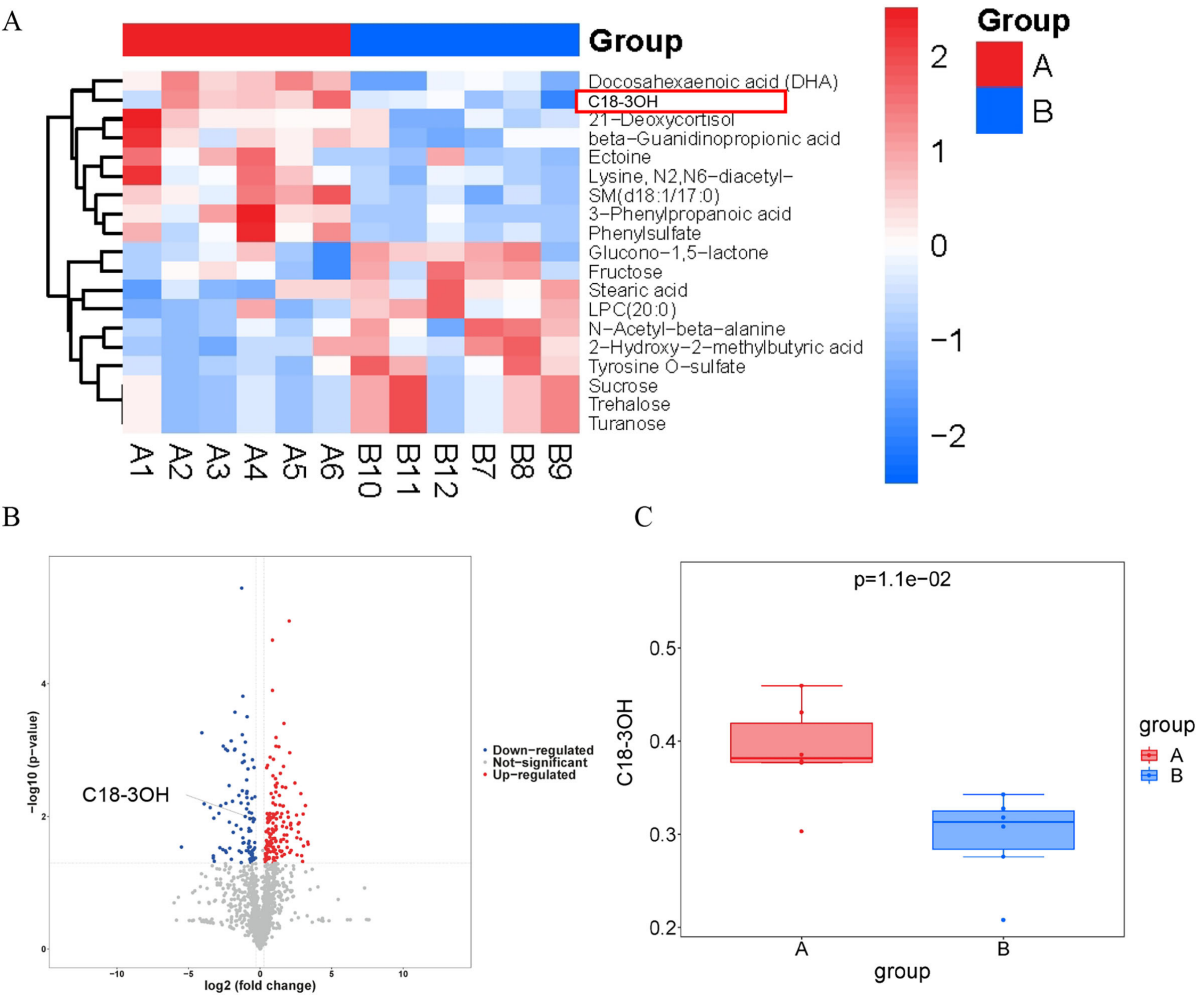
Relative content of C18-3OH in serum of apoE^-/-^ mice. **(A)** Significantly altered fatty acid metabolites in serum of apoE^-/-^ mice. **(B)** Volcano plot of altered fatty acid metabolites in serum of apoE^-/-^ mice. **(C)** Relative content of C18-3OH in serum of apoE^-/-^ mice. Group A (CD): apoE^-/-^ mice fed a chow diet. Group B (HFD): apoE^-/-^ mice fed a high-fat diet (atherosclerosis model).

C18-3OH, a fatty acid metabolite derived from gut microbiota, requires further investigation to elucidate its role in atherosclerosis. To assess the role of C18-3OH in atherosclerosis, serum levels of C18-3OH were analyzed in apoE^-/-^ mice fed either a chow diet (control) or a high-fat diet (HFD) for 12 weeks. Serum was isolated and subjected to untargeted metabolomic profiling via UHPLC-OE-MS. Results demonstrated a significant decrease in relative levels of serum C18-3OH in HFD-fed apoE^-/-^ mice compared to controls (**Figures 8A–C**). The observed reduction in serum C18-3OH levels in apoE^-/-^ mice is associated with the progression of atherosclerosis in this model.

### 3.9 Effects of C18-3OH on body weight, hepatic/renal function, and blood lipid profiles in apoE^-/-^ mice

**Figure 9:**
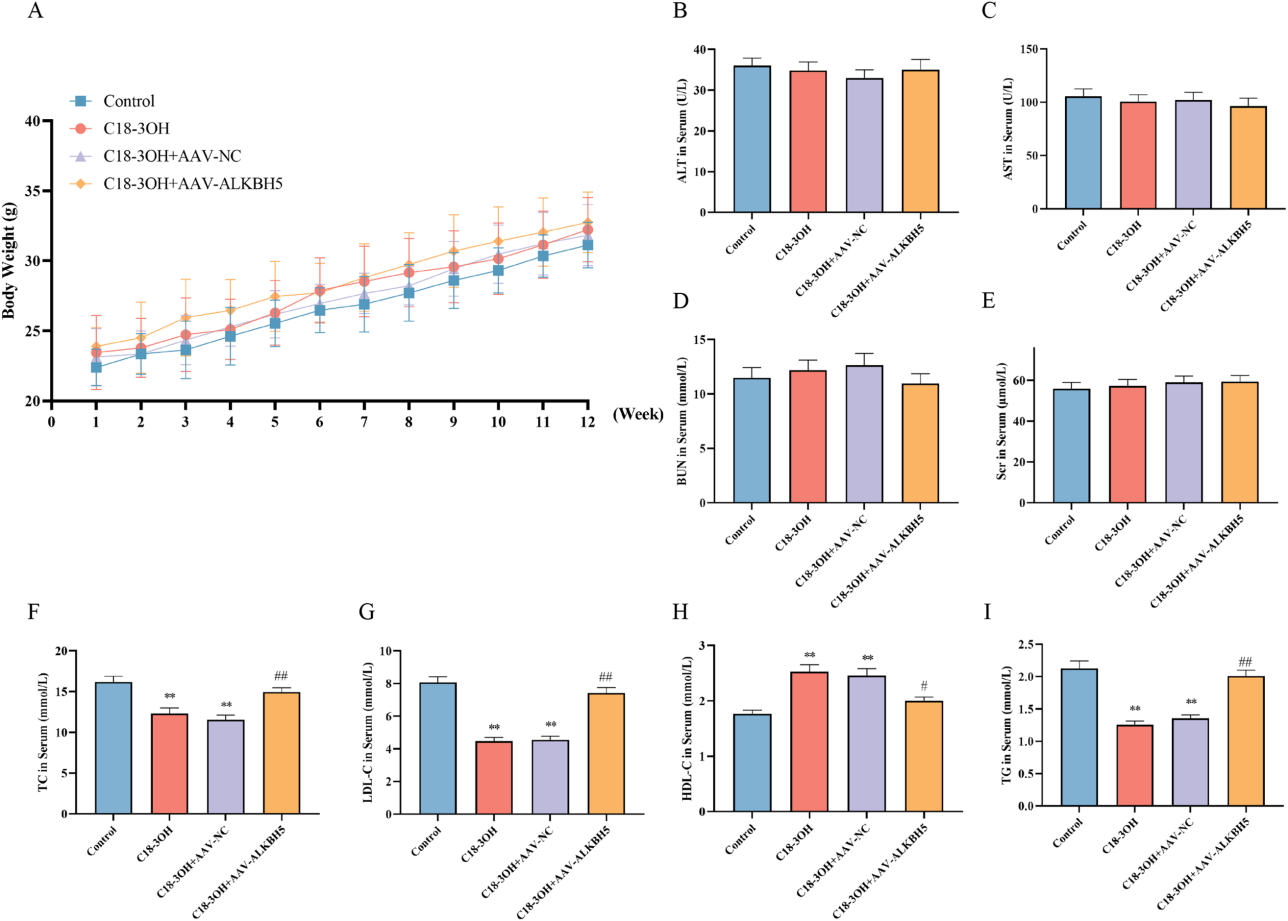
Effects of C18-3OH on body weight, hepatic/renal function, and blood lipid profiles in apoE^−/−^ mice. **(A)** Comparison of body weight among experimental groups of apoE^−/−^ mice. n=12. **(B)** Serum ALT levels in apoE^−/−^ mice. **(C)** Serum AST levels in apoE^−/−^ mice. **(D)** Serum BUN levels in apoE^−/−^ mice. **(E)** Serum Scr levels in apoE^−/−^ mice. **(F)** Serum TC levels in apoE^−/−^ mice. **(G)** Serum LDL-C levels in apoE^−/−^ mice. **(H)** Serum HDL-C levels in apoE^−/−^ mice. **(I)** Serum TG levels in apoE^−/−^ mice. ^**^P < 0.01 vs. Control; ^#^*P* < 0.05, ^##^*P* < 0.01 vs. C18-3OH + AAV-NC. n=10.

ApoE^-/-^ mice were divided into four groups: control, C18-3OH, C18-3OH + AAV-NC, and C18-3OH + AAV-ALKBH5. All groups were fed a high-fat diet (HFD) for 12 weeks, with body weight monitored periodically. Results showed progressive weight gain in all groups, but no significant intergroup differences were observed (**Figure 9A**). Hepatic and renal function were assessed by measuring serum ALT, AST, BUN, and Scr levels. No significant differences in these parameters were detected among the groups (**Figures 9B–E**), indicating that C18-3OH does not adversely affect hepatic or renal function.

Dysregulated lipid metabolism promotes the occurrence and progression of atherosclerosis. To investigate lipid metabolism, serum TC, LDL-C, HDL-C, and TG levels were analyzed. Compared to the control group, the C18-3OH and C18-3OH + AAV-NC groups exhibited significantly reduced TC, LDL-C, and TG levels, along with elevated HDL-C. However, these beneficial effects were partially reversed in the C18-3OH + AAV-ALKBH5 group, which showed increased TC, LDL-C, and TG levels and decreased HDL-C compared to the C18-3OH + AAV-NC group (**Figures 9F–I**). These findings suggest that C18-3OH ameliorates lipid metabolism in apoE^-/-^ mice, while ALKBH5 overexpression attenuates this regulatory effect.

### 3.10 C18-3OH attenuates atherosclerosis in apoE^-/-^ mice

**Figure 10:**
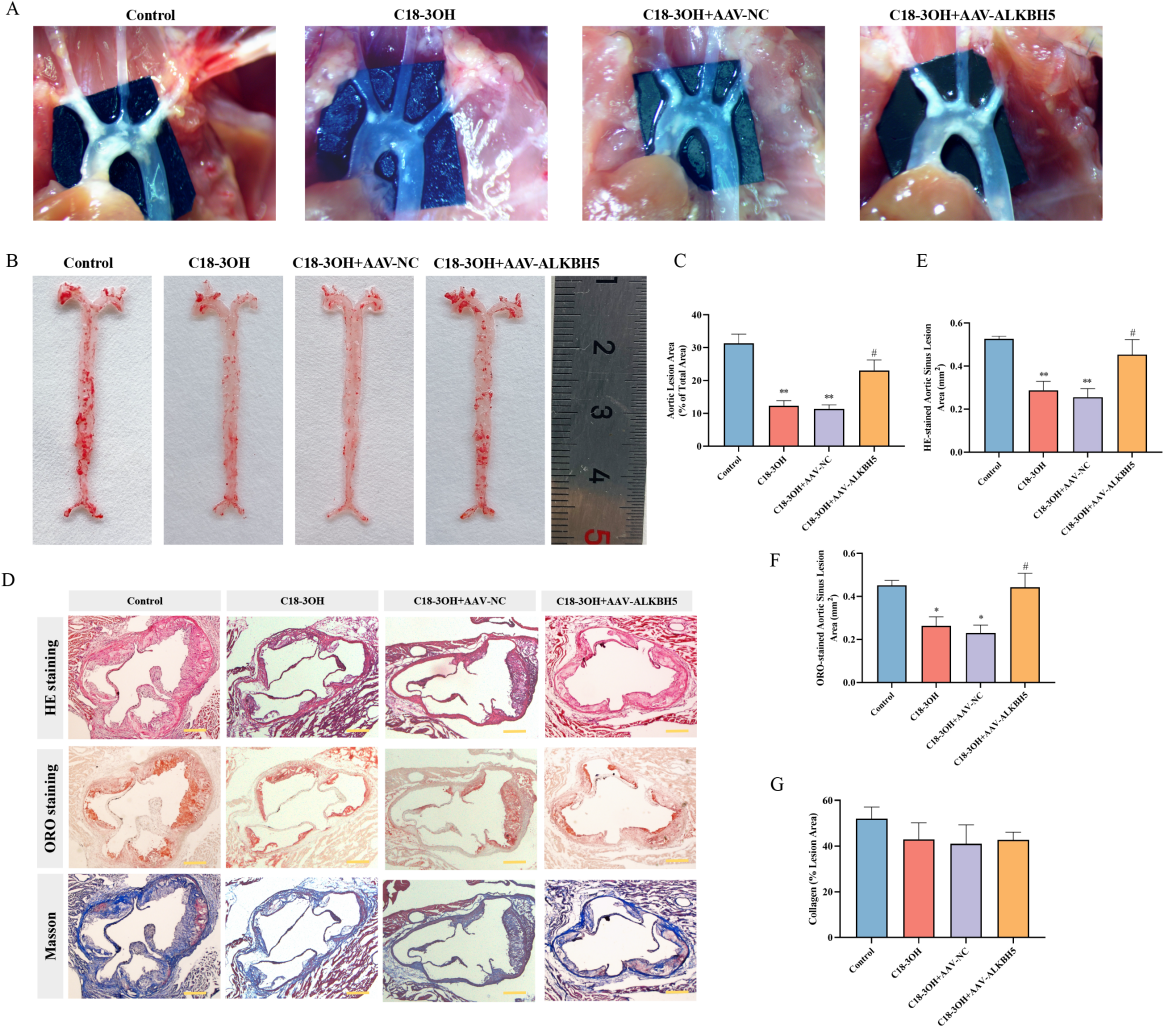
Effects of C18-3OH on atherosclerosis in apoE^-/-^ mice. **(A)** Effect of C18-3OH on atherosclerotic plaque formation in the aortic arch region of apoE^-/-^ mice. **(B)** Effect of C18-3OH on aortic wall atherosclerosis in apoE^-/-^ mice; scale bar = 4 cm. **(C)** Quantitative analysis of atherosclerotic plaque area in the aortic wall. **(D)** Effects of C18-3OH on plaque formation, lipid deposition, and collagen deposition in the aortic sinus of apoE^-/-^ mice; scale bar = 200 μm. **(E)** Quantification of aortic sinus plaque area across experimental groups. **(F)** Quantification of lipid deposition in the aortic sinus. **(G)** Quantification of collagen deposition in the aortic sinus. ^*^P < 0.05, ^**^P < 0.01 vs. control; ^#^*P* < 0.05 vs. C18-3OH + AAV-NC, n = 6.

Atherosclerotic plaque size in the aortic arch was evaluated. Compared to controls, the C18-3OH and C18-3OH + AAV-NC groups exhibited significantly reduced plaque areas, whereas the C18-3OH + AAV-ALKBH5 group showed increased plaque formation compared to the C18-3OH + AAV-NC group (**Figure 10A**). The aortic arch and its primary branches were dissected, and plaque deposition was assessed via Oil Red O staining. The C18-3OH and C18-3OH + AAV-NC groups demonstrated markedly decreased plaque areas in the aortic wall compared to controls, while the C18-3OH + AAV-ALKBH5 group displayed increased plaque accumulation compared to the C18-3OH + AAV-NC group (**Figures 10B, C**). Aortic sinus plaque composition was further analyzed using H&E, Oil Red O, and Masson’s trichrome staining. H&E staining revealed reduced atherosclerotic plaque areas in the C18-3OH and C18-3OH + AAV-NC groups compared to controls (**Figures 10D, E**). Oil Red O staining indicated decreased intraplaque lipid content in these groups (**Figures 10D–F**). Masson staining showed no significant differences in collagen content among groups (**Figures 10D–G**). Notably, the C18-3OH + AAV-ALKBH5 group exhibited increased aortic sinus plaque burden compared to the C18-3OH and C18-3OH + AAV-NC groups. These results demonstrate that C18-3OH reduces atherosclerotic plaque area via ALKBH5 regulation.

### 3.11 Effects of C18-3OH on ABCA1, PAX-8, ALKBH5 expression, and PAX-8 mRNA m^6^A methylation in aortas of apoE^-/-^ mice

**Figure 11:**
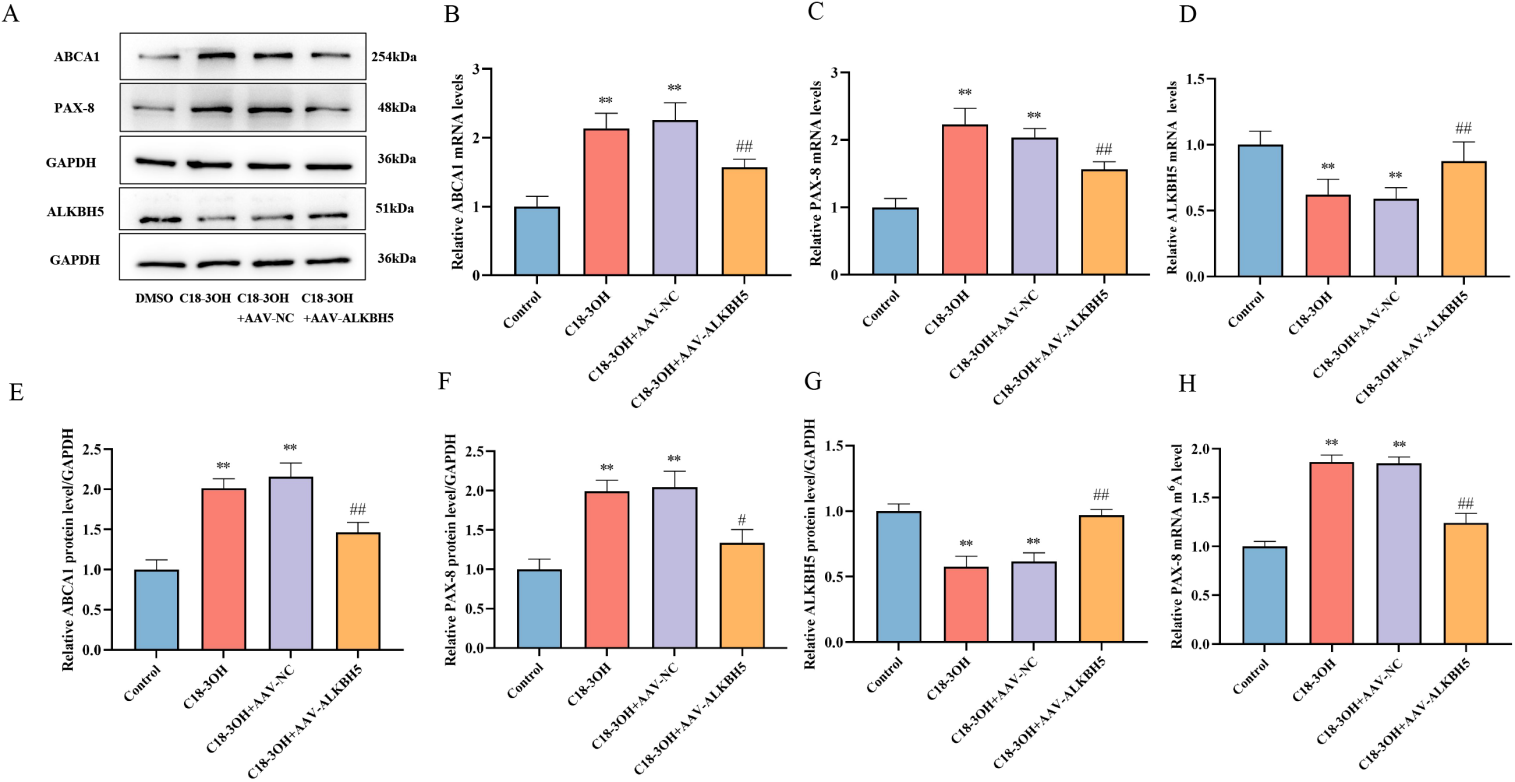
Effects of C18-3OH on ABCA1, PAX-8, ALKBH5 expression, and PAX-8 mRNA m^6^A methylation in Aortas of apoE^−/−^ Mice. **(A)** Western blot analysis of ABCA1, PAX-8, and ALKBH5 protein expression in aortas of HFD-fed apoE^−/−^ mice treated with C18-3OH and/or ALKBH5 overexpression. **(B)** RT-qPCR analysis of ABCA1 mRNA expression in aortas of apoE^−/−^ mice. **(C)** RT-qPCR analysis of PAX-8 mRNA expression in aortas of apoE^−/−^ mice. **(D)** RT-qPCR analysis of ALKBH5 mRNA expression in aortas of apoE^−/−^ mice. **(E)** Quantitative analysis of ABCA1 protein expression (Panel A). **(F)** Quantitative analysis of PAX-8 protein expression (Panel A). **(G)** Quantitative analysis of ALKBH5 protein expression (Panel A). **(H)** MeRIP-qPCR analysis of PAX-8 mRNA m^6^A methylation levels in aortas of apoE^−/−^ mice across experimental groups. ^**^P < 0.01 vs. Control; ^#^*P* < 0.05, ^##^*P* < 0.01 vs. C18-3OH + AAV-NC. n=6.

RT-qPCR and Western blot analyses were performed to assess mRNA and protein levels of ALKBH5, PAX-8, and ABCA1 in aortic tissues. Compared to the control group, the C18-3OH and C18-3OH + AAV-NC groups exhibited significant upregulation of PAX-8, ABCA1 mRNA, and protein expression, alongside downregulation of ALKBH5 mRNA and protein expression. In contrast, the C18-3OH + AAV-ALKBH5 group showed reduced PAX-8 and ABCA1 expression and increased ALKBH5 expression compared to the C18-3OH + AAV-NC group (**Figures 11A–G**), indicating that C18-3OH regulates PAX-8 and ABCA1 expression *in vivo* via ALKBH5.

To evaluate PAX-8 mRNA m^6^A modification levels and their association with ALKBH5, MeRIP-qPCR was conducted. The C18-3OH and C18-3OH + AAV-NC groups displayed elevated PAX-8 mRNA m^6^A methylation compared to controls, whereas the C18-3OH + AAV-ALKBH5 group showed reduced methylation relative to the C18-3OH + AAV-NC group (**Figure 11H**). These results demonstrate that C18-3OH enhances PAX-8 mRNA m^6^A modification *in vivo* through ALKBH5 regulation.

## 4 Discussion

Gut microbiota-derived bioactive metabolites modulate host physiological functions through genetic and epigenetic mechanisms, directly or indirectly influencing the development of cardiovascular diseases such as coronary artery disease, heart failure, hypertension, and obesity (36). The relationship between gut microbial metabolites and lipid metabolism has received increasing attention (37). Fatty acid metabolites from gut microbiota represent a novel class of mediators in cardiovascular pathophysiology. Fatty acids are categorized into three types: short-chain fatty acids (<6 carbons), medium-chain fatty acids (6–12 carbons), and long-chain fatty acids (>12 carbons). Short-chain fatty acids exert significant regulatory effects on lipid metabolism and atherosclerosis by modulating macrophage inflammation, cholesterol metabolism, and endothelial function (38). However, the roles and mechanisms of long-chain fatty acids in lipid metabolism and atherosclerosis remain incompletely characterized.

Foam cell accumulation and subsequent plaque formation constitute a critical pathological basis for atherosclerosis. Maintenance of cellular cholesterol homeostasis is essential for normal cellular function, as cholesterol accumulation plays a key role in the pathogenesis of foam cell. Cholesterol efflux serves as a primary pathway for peripheral tissues to eliminate excess cholesterol from foam cells. ABCA1, the most pivotal transporter mediating reverse cholesterol transport (RCT) in atherosclerosis, plays a central role in this process (39, 40). Microbial metabolites are increasingly recognized as regulators of macrophage cholesterol metabolism. For instance, the tryptophan metabolite indole-3-propionic acid (IPA) promotes cholesterol efflux via ABCA1 upregulation, thereby attenuating coronary artery disease progression; hence, IPA is negatively correlated with the risk of atherosclerosis (41). Similarly, the gut microbiota-derived butyrate ameliorates atherosclerosis in apoE^-/-^ mice through ABCA1-mediated cholesterol efflux (42). In contrast, TMAO exacerbates macrophage lipid accumulation and atherosclerosis by suppressing ABCA1 expression (43). C18-3OH, a long-chain fatty acid metabolite, remains poorly characterized in lipid metabolism. C18-3OH is associated with inflammatory diseases and is involved in the regulation of obesity and insulin resistance (20, 21). This suggests that C18-3OH may regulate atherosclerosis. In this study, treatment of foam cells with C18-3OH significantly reduced intracellular cholesterol content, as evidenced by decreased lipid droplet accumulation in Oil Red O-stained cells, suggesting its inhibitory effect on foam cell lipid deposition. Mechanistically, C18-3OH upregulated ABCA1 expression in foam cells, enhancing cholesterol efflux to the apoA-I receptor. These findings collectively demonstrate that the gut microbiota-derived metabolite C18-3OH suppresses foam cell lipid accumulation by promoting ABCA1-dependent cholesterol efflux.

Transcription factors play pivotal roles in regulating gene expression by specifically recognizing and binding to target DNA sequences to modulate transcriptional processes. Multiple transcription factors have been identified to regulate ABCA1 expression through direct or indirect mechanisms. For instance, the transcriptional repressor sterol response element-binding protein suppresses ABCA1 expression by binding to E-box motifs within the ABCA1, thereby accelerating atherosclerosis progression in apoE^-/-^ mice (44). Liver X receptor and peroxisome proliferator-activated receptor participate in lipid metabolism regulation by modulating ABCA1 transcription (45). PAX-8 has been implicated in apoptosis, adipocyte transformation, and angiogenesis (23). However, the role of PAX-8 in atherosclerosis and foam cell formation remains unexplored. This study demonstrated that PAX-8 upregulation promotes ABCA1 expression in foam cells. C18-3OH treatment significantly increased both PAX-8 and ABCA1 expression, while PAX-8 knockdown attenuated C18-3OH-induced ABCA1 upregulation and impaired cholesterol efflux. These findings establish that C18-3OH enhances ABCA1 expression and cholesterol efflux in foam cells through PAX-8-dependent regulation.

RNA modifications dynamically regulate gene expression to maintain cellular homeostasis and function. m^6^A is a pivotal post-transcriptional modification. Abnormal m^6^A modification has been implicated in vascular inflammation, oxidative stress, lipid metabolism dysregulation, and atherosclerotic plaque destabilization (46, 47). By modulating RNA splicing, translation, and stability, m^6^A modification contributes to the pathogenesis of atherosclerosis-related conditions, including cardiovascular diseases and stroke (48, 49). Bioinformatic analysis identified multiple m^6^A modification sites within the genomic sequences of human and murine PAX-8 mRNA. In this study, C18-3OH significantly enhanced m^6^A modification levels of PAX-8 mRNA in foam cells. Thus, C18-3OH may upregulate PAX-8 expression by increasing PAX-8 mRNA m^6^A methylation.

ALKBH5 plays critical roles in cardiovascular diseases, including ischemia/reperfusion injury, valvular heart disease, and cardiac hypertrophy (50–52). Studies indicate that ALKBH5 aids in the diagnosis of acute myocardial infarction, predicts its risk, and modulates immune cell infiltration, ferroptosis, and oxidative stress (31). These findings suggest that ALKBH5 may be involved in atherosclerosis development. In the cardiovascular system, ALKBH5 plays different roles in specific cell types. For instance, macrophage-specific ALKBH5 knockout attenuates Ang II-induced macrophage-to-myofibroblast transition, thereby ameliorating myocardial fibrosis and dysfunction (53). ALKBH5 also regulates ferroptosis and macrophage inflammation via m^6^A-dependent mechanisms (54, 55). However, its role in macrophage lipid metabolism and atherosclerosis remained undefined. To delineate the contribution of ALKBH5 to C18-3OH-mediated regulation of ABCA1 expression, lentiviral transfection was employed to overexpress ALKBH5. Results demonstrated that ALKBH5 overexpression partially reversed C18-3OH-induced enhancement of PAX-8 mRNA m^6^A methylation, PAX-8/ABCA1 expression, and cholesterol efflux. ChIP-Seq and ChIP-qPCR confirmed direct binding of the transcription factor PAX-8 to the ABCA1, establishing its regulatory role in ABCA1 expression. Collectively, these data indicate that C18-3OH suppresses ALKBH5 activity, thereby increasing PAX-8 mRNA m^6^A modification, upregulating PAX-8 and ABCA1 expression, and enhancing cholesterol efflux.

The gut microbiota functions as an endocrine organ, generating bioactive metabolites that influence host physiology (56). Microbial modulation of cholesterol and lipid metabolism significantly impacts atherosclerotic plaque formation (57). C18-3OH, a long-chain fatty acid metabolite, was investigated in this study. Untargeted metabolomics revealed significantly reduced serum C18-3OH levels in HFD-fed apoE^-/-^ mice compared to controls. C18-3OH administration markedly attenuated aortic atherosclerotic plaque area in HFD-fed apoE^-/-^ mice, suggesting its anti-atherogenic potential. Dysregulated lipid metabolism is a major risk factor for cardiovascular diseases, particularly atherosclerosis and coronary artery disease. Lipid metabolism disorders in the serum directly or indirectly elevate cardiovascular morbidity and mortality (58). Elevated LDL and TG levels increase the risk of atherosclerotic cardiovascular events (59), whereas HDL exhibits an inverse correlation with disease progression. HDL confers athero-protection primarily via reverse cholesterol transport, facilitating cholesterol efflux from arterial macrophages to the liver for excretion. In this study, C18-3OH-treated mice exhibited higher serum HDL-C levels and lower LDL-C, TC, and TG levels compared to HFD-fed atherosclerotic controls, demonstrating the capacity of C18-3OH to improve lipid homeostasis and counteract atherosclerosis. Furthermore, C18-3OH did not alter serum AST, ALT, BUN, or Scr levels, indicating no hepatotoxic or nephrotoxic effects.

m^6^A methylation contributes to atherosclerosis pathogenesis by regulating programmed cell death, macrophage inflammation, endothelial cell injury, and smooth muscle cell phenotypic switching (60–62). In this study, C18-3OH treatment downregulated ALKBH5 expression in the aortas of apoE^-/-^ mice while increasing PAX-8 mRNA m^6^A methylation and PAX-8, ABCA1 mRNA, and protein expression, thereby attenuating atherosclerotic progression. Notably, the anti-atherogenic and lipid-modulatory effects of C18-3OH were partially reversed by ALKBH5 overexpression.

These findings demonstrate that C18-3OH enhances reverse cholesterol transport and ameliorates lipid metabolism and atherosclerosis in apoE^-/-^ mice via the ALKBH5/PAX-8/ABCA1 axis. This study provides novel insights into the role of gut microbiota-derived metabolites in atherosclerotic cardiovascular diseases, suggesting C18-3OH as a potential therapeutic strategy. A comprehensive investigation of m^6^A methylation mechanisms will enhance our understanding of atherosclerotic pathogenesis, highlighting m^6^A methylation as a promising therapeutic target for atherosclerosis.

## 5 Conclusion

The gut microbiota-derived metabolite, C18-3OH, suppresses the demethylase ALKBH5, thereby enhancing PAX-8 mRNA m^6^A methylation and PAX-8 expression. This upregulates ABCA1, promoting cholesterol efflux from THP-1 macrophage-derived foam cells and reducing intracellular lipid accumulation. Furthermore, C18-3OH ameliorates lipid metabolism and attenuates atherosclerosis in apoE^-/-^ mice.

## Data Availability

The ChIP-seq data have been deposited in the GEO repository under accession number GSE317586.
